# Honor to the Brave

**Published:** 1864-11

**Authors:** 


### HONOR TO THE BRAVE.

Not many weeks ago, a band of twelve physicians, occupying various positions in the army stationed in Canada, left our city (under orders from the authorities) for Bermuda,—then and still the scene of fearful ravages from yellow fever. Few who bade them farewell, and who knew the fearful fatality of the epidemic they were about to encounter, ever imagined that all would pass the ordeal unscathed. Too soon has this fear been realized. Hardly had this devoted band landed upon the pestilential shores, and entered upon the discharge of their duties, than one of their number was prostrated by the disease. Poor Milroy, the active, energetic assistant surgeon of the 30th Regiment, now stationed in this garrison, was the first victim. Not long was he allowed to labor on his noble mission, ere he passed away, a victim to the disease he went so far to assist in arresting.

At last accounts the disease was raging with unabated fury, and those able were leaving the country. Who can tell upon whom the fell destroyer will pounce as his next victim? for, worn out by watching, dispirited by want of success, they are indeed apt to contract the disease. Who but will remember the fearful epidemic of yellow fever at Norfolk, Virginia, in 1855, when forty physicians fell in the hopeless contest? God grant this visitation in Bermuda may at its close give no such list. How few think of the dangers which the profession is exposed in the discharge of its duties. How few, when they heard of the departure of the twelve physicians for Bermuda, even thought of the dangers they would so soon meet; and yet they are as great as that encountered by assistant-surgeons Manley and Temple in their brave conduct at the recent engagements in New Zealand, and for which the Queen has decorated them with that badge of distinguished bravery, the Victoria Cross. We cannot but admire the spirit of true heroism which is exhibited by the man who, at the call of duty, walks to almost cestain death, in aid of his fellow-creatures, suffering from a malignant infectious disease; this, in our opinion, is of greater merit, than he who marches to the cannon's mouth, during moments of intense excitement. One exhibits the cool, collected determination, self-sacrificing benevolence of the Christian; the other the plucky spirit and gallantry of a brave man,—both equally to be admired, and equally deserving of recognition. But if no outward decoration is worn on the breast of the army or civilian physician who so often breathes the pestilential air so filled with summonses of death, there is that inward satisfaction which every physician feels when he knows that he is simply doing his duty, and exerting the talents which his Creator may have given him for the benefit of his suffering fellow-creatures,—*Canada Medical Journal.*

